# Incomplete endothelialization of an atrial septal occluder suggested by multimodality imaging

**DOI:** 10.1093/ehjcr/ytag583

**Published:** 2026-08-03

**Authors:** Hezhi Li, Hongwen Fei, Jimei Chen, Caojin Zhang

**Affiliations:** Guangdong Cardiovascular Institute, Guangdong Provincial People's Hospital, Guangdong Academy of Medical Sciences, 106 Zhongshan 2nd Road, Guangzhou,Guangdong Province 510080, China; Department of Ultrasound, Guangdong Provincial People’s Hospital (Guangdong Academy of Medical Sciences), Southern Medical University, Guangzhou, Guangdong Province 510080, China; Guangdong Cardiovascular Institute, Guangdong Provincial People's Hospital, Guangdong Academy of Medical Sciences, 106 Zhongshan 2nd Road, Guangzhou,Guangdong Province 510080, China; Department of Cardiology, Guangdong Provincial People’s Hospital (Guangdong Academy of Medical Sciences), Southern Medical University, 106 Zhongshan 2nd Road, Guangzhou, Guangdong Province 510080, China

**Keywords:** Atrial septal defect, Device closure, Incomplete endothelialization, Multimodality imaging, Interventional cardiology

## Summary

A 45-year-old woman had persistent intra-device flow and thrombus 6 months after atrial septal defect closure. Multimodality imaging showed partial embedding of the left atrial disc within the right lower pulmonary vein ostium, constraining expansion. Relative device oversizing and pulmonary venous flow exposure may have hindered endothelial coverage.

## Case description

**Figure 1:**
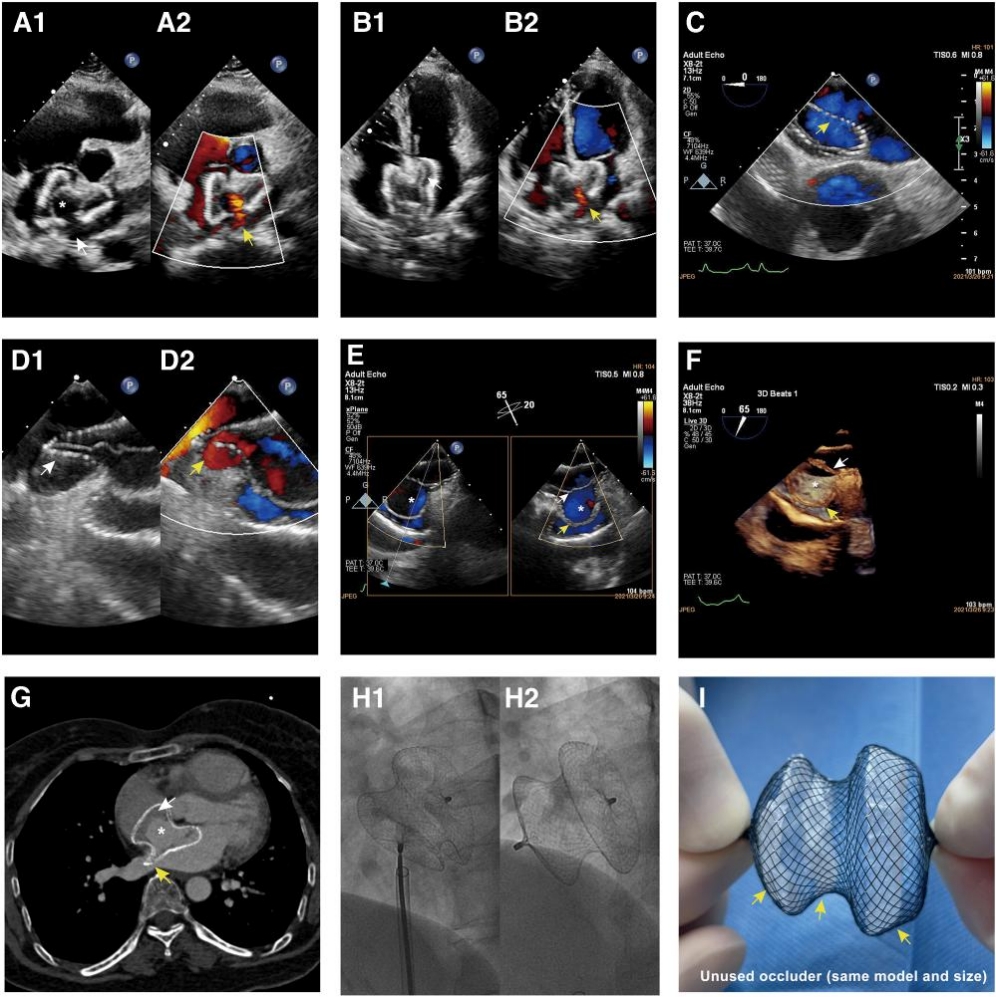
Multimodality imaging and procedural assessment of an atrial septal occluder with constrained expansion and suspected incomplete endothelialization. (*A–B*) Transthoracic echocardiography: (**a1**) parasternal long-axis view showing a patent intra-device cavity (*) and abnormal device contour near the right lower pulmonary vein (white arrow); (**a2**) colour Doppler showing inflow into the cavity (yellow arrow); (**b1**) apical four-chamber view showing oscillation of an internal membrane (white arrow); and (**b2**) colour Doppler showing bidirectional intra-device flow (yellow arrow). (*C–D*) Transoesophageal echocardiography: (*C*) flow across the left atrial disc (white arrow); (**d1**) deformation of the left atrial disc (white arrow); and (**d2**) colour Doppler inflow at the deformed region (yellow arrow). (*E–F*) X-plane and three-dimensional transoesophageal echocardiography showing the patent intra-device cavity (*) and internal membranes (white and yellow arrows). (*G*) Computed tomography angiography showing partial embedding of the left atrial disc within the right lower pulmonary vein ostium (yellow arrow), constrained expansion, device-associated thrombus (white arrow), and contrast pooling (*). (*H*) Intra-procedural fluoroscopy: (**h1**) device configuration immediately before release while the occluder remained attached to the delivery cable; (**h2**) device configuration immediately after release. **(***I***)** An unused Cardi-O-Fix ASD Occluder of the same model, shown ex vivo to illustrate its normal internal architecture. Yellow arrows indicate the three internal occlusive membranes. The device shown was not explanted from the patient. Abbreviation: ASD, atrial septal defect; TEE, transoesophageal echocardiography; TTE, transthoracic echocardiography.

At 6-month follow-up, transthoracic echocardiography (TTE) showed a patent intra-device cavity with inflow, membrane oscillation, and bidirectional flow (*Figure 1A* and *B*; Supplementary material online, *Video S1*). Transoesophageal echocardiography (TEE), including X-plane and three-dimensional imaging, delineated disc deformation, cavity, and membranes (*Figure 1C–F*; Supplementary material online, *Video S2*). Computed tomography angiography showed the left atrial disc partly embedded in the right lower pulmonary vein ostium, with constrained expansion, contrast pooling, and thrombus but no significant pulmonary vein stenosis (*Figure 1G*).

Pre-procedural TTE showed an elliptical 29 × 20-mm defect, a 59-mm septal length, and a 14-mm distance to the right pulmonary vein. Neither TEE nor balloon sizing was performed. After a multipurpose angiographic catheter crossed the defect, repeat TTE guided implantation of a 42-mm Cardi-O-Fix ASD Occluder with 58- and 42-mm left- and right-atrial discs. It exceeded the maximum defect diameter by 13 mm. Fluoroscopy showed initial deployment, recapture/redeployment, full deployment with the cable attached, and release (*Figure 1H1–h2*; Supplementary material online, *Video S3*). The operative record noted no immediate residual shunt. An unused same-model occluder showed three occlusive membranes (*Figure 1I*).

After implantation, aspirin and clopidogrel were given for 1 month, followed by aspirin alone for 5 months. Experimental studies have demonstrated progressive endothelial coverage after device implantation.^1,2^ The 58-mm left disc nearly matched septal length. Partial embedding restricted expansion, and continued exposure to pulmonary venous flow may have impeded endothelial coverage and promoted thrombus formation. Surgery was recommended but declined. No further antiplatelet or anticoagulant therapy was documented. At 2-year echocardiography, device morphology and intra-device flow were unchanged; thrombus resolution was unconfirmed.

## Supplementary Material

ytag583_Supplementary_Data

## Data Availability

No new data were generated or analysed in support of this research.

## Notes

**Consent:** The authors confirm that written consent for submission and publication of this case report, including images and associated text, has been obtained from the patient in line with COPE guidance.
