# Supplementary material for: Incomplete endothelialization of an atrial septal occluder suggested by multimodality imaging
Source: Eur Heart J Case Rep. 2026 Aug 3;10(8):ytag583. doi: 10.1093/ehjcr/ytag583 (PMC13484012; doi:10.1093/ehjcr/ytag583)
Supplement: Supplementary file 1 [file ytag583_supplementary_data.zip › Video Legends.docx]

**Inline Video Legends**

**Video 1. Multiplane transthoracic echocardiographic assessment of the atrial septal occluder.** The first segment shows an abnormal patent intra-device cavity and loss of normal device structure adjacent to the right pulmonary vein in the parasternal view. The second segment shows blood flow entering the cavity from the right lower margin on colour Doppler imaging. The third segment shows oscillation of an internal membrane within the left atrial disc in the apical four-chamber view, and the fourth segment demonstrates bidirectional intra-device flow on colour Doppler imaging.

**Video 2. Multiplanar and three-dimensional transoesophageal echocardiographic assessment of the atrial septal occluder.** Sequential segments demonstrate trans-device flow through the left atrial disc mesh, deformation and constrained expansion of the left atrial disc, blood flow entering the intra-device cavity at the deformed region, a patent cavity with internal flow on X-plane imaging, and the intra-device cavity with visible internal occlusive membranes on three-dimensional transoesophageal echocardiography.

**Video 3. Intra-procedural fluoroscopy showing the occluder deployment and release sequence.** Sequential segments demonstrate initial deployment of the left atrial disc, partial recapture and redeployment, full deployment of both atrial discs with the device still attached to the delivery cable, and final release of the device from the delivery cable.
